# Potential Formula for the Calculation of Starting and Incremental Insulin Glargine Doses: ALOHA Subanalysis

**DOI:** 10.1371/journal.pone.0041358

**Published:** 2012-08-01

**Authors:** Takashi Kadowaki, Tetsuya Ohtani, Masato Odawara

**Affiliations:** 1 Department of Diabetes and Metabolic Diseases, Graduate School of Medicine, The University of Tokyo, Tokyo, Japan; 2 sanofi-aventis K.K., Tokyo, Japan; 3 Department of Diabetology, Metabolism, and Endocrinology, Tokyo Medical University, Tokyo, Japan; Tehran University of Medical Sciences, Islamic Republic of Iran

## Abstract

**Background:**

Pragmatic methods for dose optimization are required for the successful basal management in daily clinical practice. To derive a useful formula for calculating recommended glargine doses, we analyzed data from the Add-on Lantus® to Oral Hypoglycemic Agents (ALOHA) study, a 24-week observation of Japanese type 2 diabetes patients.

**Methodology/Principal Findings:**

The patients who initiated insulin glargine in basal-supported oral therapy (BOT) regimen (n = 3506) were analyzed. The correlations between average changes in glargine dose and HbA1c were calculated, and its regression formula was estimated from grouped data categorized by baseline HbA1c levels. Starting doses of the background-subgroup achieving the HbA1c target with a last-observed dose above the average were compared to an assumed optimal starting dose of 0.15 U/kg/day. The difference in regression lines between background-subgroups was examined. A formula for determining the optimal starting and titration doses was thereby derived. The correlation coefficient between changes in dose and HbA1c was −0.9043. The estimated regression line formula was −0.964 × change in HbA1c+2.000. A starting dose of 0.15 U/kg/day was applicable to all background-subgroups except for patients with retinopathy (0.120 U/kg/day) and/or with eGFR<60 mL/min/1.73 m^2^ (0.114 U/kg/day). Additionally, women (0.135 U/kg/day) and patients with sulfonylureas (0.132 U/kg/day) received a slightly decreased starting dose.

**Conclusions/Significance:**

We suggest a simplified and pragmatic dose calculation formula for type 2 diabetes patients starting glargine BOT optimal daily dose at 24 weeks  =  starting dose (0.15×weight) + incremental dose (baseline HbA1c − target HbA1c+2). This formula should be further validated using other samples in a prospective follow-up, especially since several patient groups required lower starting doses.

## Introduction

Type 2 diabetes mellitus is a progressive disease characterized by insulin insufficiency and resistance with chronic hyperglycemia. In the early stage, oral antidiabetic drugs (OADs) are typically selected as the initial intervention. However, higher doses or additional medications are ultimately required in many cases to reach blood sugar target levels. Generally, OADs are effective only for a limited time [1], with a majority of patients eventually requiring insulin therapy. One typical approach to insulin initiation is to add once-a-day basal insulin, such as insulin glargine, while maintaining the previous OAD regimen [2].

Insulin therapy has the optimal glucose lowering effect when used at appropriate dosages. The insulin dose should be continuously adjusted until the glycemic control target is reached. In the Treat-To-Target paradigm, adequate insulin doses at initiation and appropriative titration of doses, based on fasting plasma glucose (FPG) measurement, are desirable for managing blood sugar levels [3]. At initiation, a common starting dose is 0.2 U/kg/day or 10.0 U/day in western countries [2]. Regarding incremental doses, numerous studies conducted on western populations have shown patients treated with basal insulin at forced-titration doses according to FPG-monitored algorithms are more often achieving the target HbA1c [3]. Thus, an algorithm based on FPG-monitoring has been recommended for determining optimal starting and incremental (typically, 2 units every 3 days until fasting levels are consistently within target range [70–130 mg/dL]) doses of insulin [2].

**Figure 1:**
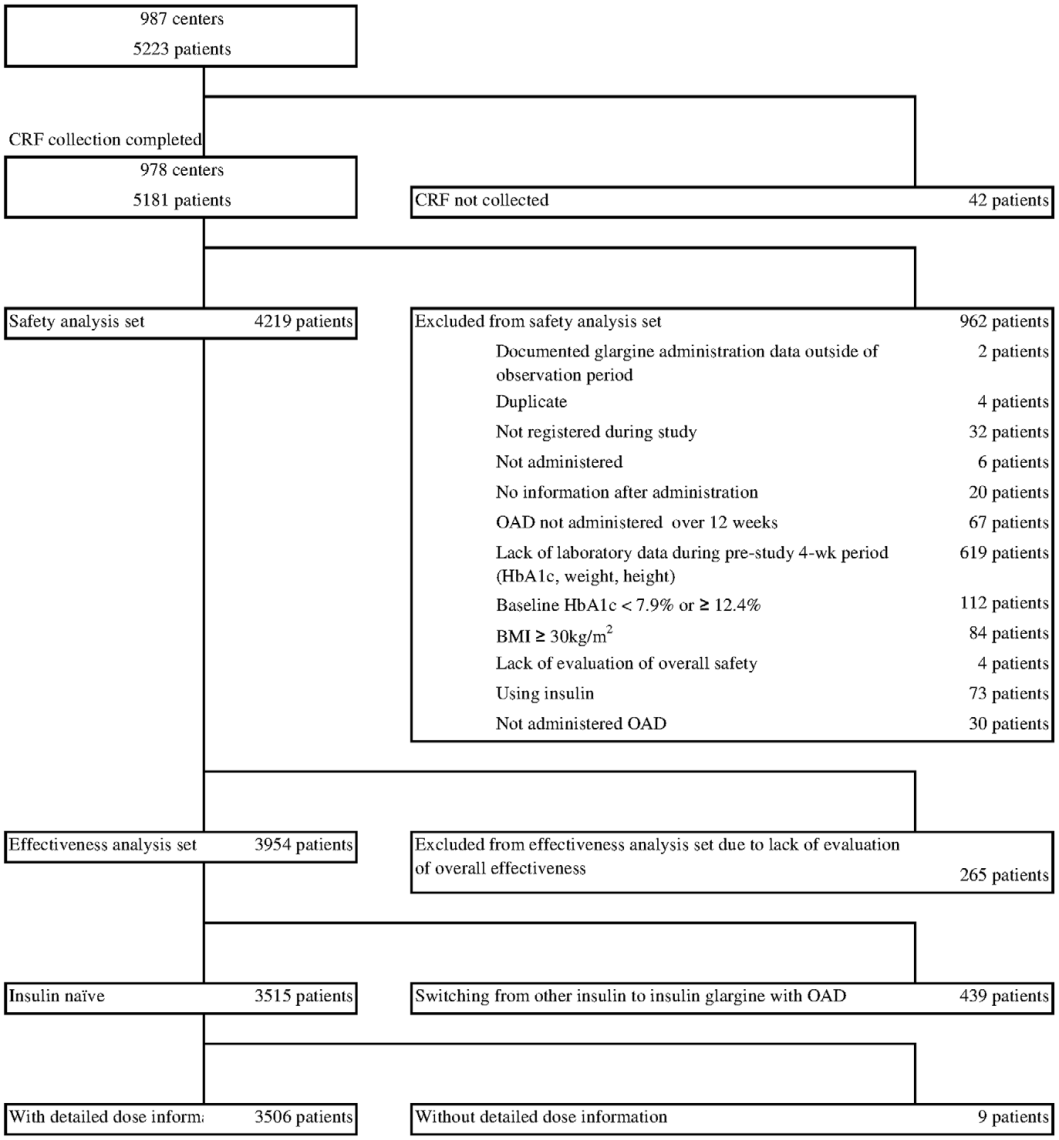
Disposition of patients.

The practice of starting insulin therapy with a dose of 0.2 U/kg/day or 10.0 U/day and application of the FPG-monitored titration algorithm do not, however, appear to be widespread in Japan. For example, the average daily dose at 24 weeks after insulin initiation was less than 10.0 U/day, and less than 20% of patients achieved HbA1c levels of 7.0% or lower in the Add-on Lantus® to Oral Hypoglycemic Agents (ALOHA) study: a 24-week, prospective, open-label, multicenter, observational assessment of the safety and effectiveness of basal supported oral therapy (BOT) with insulin glargine for treating Japanese patients with type 2 diabetes in a routine clinical setting [4].

Insufficient dose adjustment in Japanese type 2 diabetes has two possible explanations. One involves inapplicability of findings from western populations to the Japanese population due to potential ethnic differences in pathophysiology of type 2 diabetes: body anthropometry, insulin secretion capacity, contribution of insulin resistance, etc [5]–[7]. Another may involve the impracticality; frequent FPG monitoring discourages physicians and patients from performing adequate titration, suggesting a need for a simple titration guide that can easily be employed in routine clinical care.

To address these issues, we aimed to generate a simple and pragmatic formula using an individual patient’s HbA1c and weight, for determining appropriate starting and incremental doses of insulin glargine in BOT for Japanese type 2 diabetes.

## Methods

### Study Design and Patients

The ALOHA study was conducted as a post-marketing surveillance of insulin glargine use between 2007 and 2009 in 987 hospitals and clinics throughout Japan. The study results were reported in detail previously [4]. Japanese patients with type 2 diabetes requiring insulin therapy were eligible for documentation in the ALOHA study. All eligible patients satisfied the following criteria during a 4-week screening period: 1) previously treated with OAD(s) for at least 12 weeks, 2) HbA1c ≥7.9% and <12.5% (These values were originally selected based on the Japan Diabetes Society [JDS] values [≥7.5% and <12.0%, respectively]). HbA1c data were collected as JDS values, and then converted to National Glycohemoglobin Standardization Program (NGSP) values by the following conversion formula: HbA1c (NGSP) = 1.02×HbA1c (JDS)+0.25% [8]. NGSP values were used herein, 3) body mass index (BMI) (weight in kilograms divided by the square of height in meters) ≤30.

**Table 1:** Change in glargine dose, dose per weight, and HbA1c.

Baseline HbA1c	Δ Dose		Δ Dose/Weight		Δ HbA1c	
	n	Mean±SD	n	Mean±SD	n	Mean±SD
Overall	3506	3.5±4.8	2622	0.056±0.076	3187	−1.53±1.23
7.9≤HbA1c<8.5	775	2.6±3.9	565	0.044±0.066	701	−0.85±0.67
8.5≤HbA1c<9.0	580	3.0±4.3	432	0.049±0.069	529	−1.04±0.76
9.0≤HbA1c<9.5	526	3.5±4.5	403	0.055±0.069	478	−1.29±0.89
9.5≤HbA1c<10.0	483	4.0±4.9	358	0.062±0.073	445	−1.52±1.08
10.0≤HbA1c<10.5	330	4.0±4.5	246	0.063±0.071	295	−1.77±1.14
10.5≤HbA1c<11.0	303	4.2±5.1	221	0.064±0.075	268	−2.19±1.18
11.0≤HbA1c<11.5	197	4.2±6.2	153	0.068±0.105	186	−2.58±1.38
11.5≤HbA1c<12.0	152	4.1±5.5	117	0.061±0.087	136	−2.77±1.56
12.0≤HbA1c<12.5	160	5.0±6.7	127	0.078±0.108	149	−3.20±1.75

**Figure 2:**
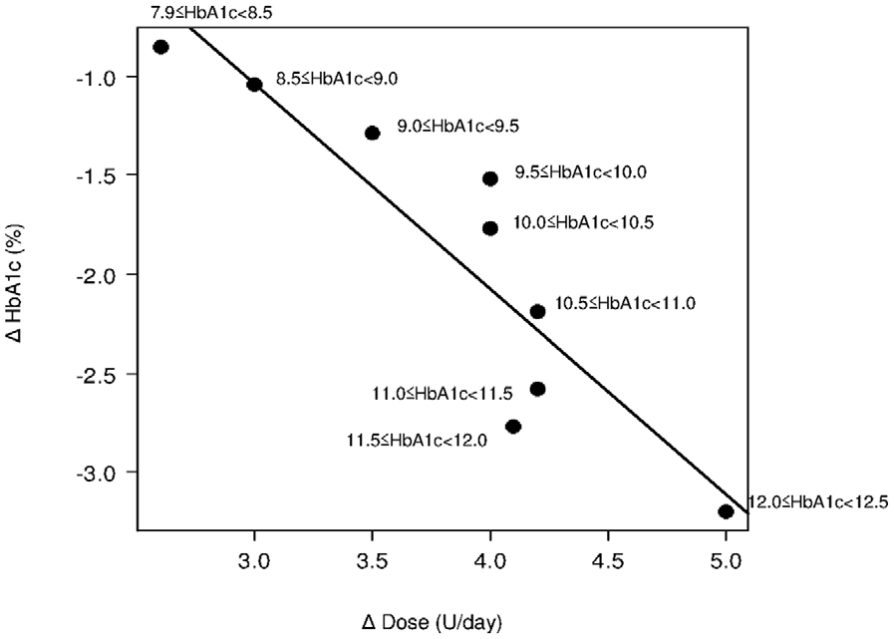
Plots and regression line describing the association between changes in dose and HbA1c stratified by baseline HbA1c levels. r = −0.9043; Adjusted R^2^ = 0.7918 Δ HbA1c = −1.037×Δ Dose+2.074 Δ Dose = −0.964×Δ HbA1c+2.000 Δ Dose ≈ − Δ HbA1c+2 =  (Baseline HbA1c - Target HbA1c)+2.

To briefly summarize the results of the ALOHA study [4], 5223 patients in total were enrolled, and safety and effectiveness data were available for 4219 and 3954 patients, respectively. Patient age in the safety assessment population (mean ± SD) was 62.8±12.1 years, and 2485 patients (58.9%) were male. As to safety, 44 patients experienced hypoglycemic episodes (43 with symptomatic, including 2 with severe and 2 with loss of consciousness [4 cases with severe hypoglycemia (0.1%)], and 1 with asymptomatic hypoglycemia detected by the attending physician). As to effectiveness, the reductions in mean HbA1c, FPG, and PPG from baseline were 1.43±1.23%, 59.71±65.93 mg/dL, and 60.72±92.42 mg/dL, respectively (P<0.0001 for all; paired *t*-test). The mean weight increase from baseline was 0.83±2.52 kg (P<0.0001; paired *t*-test).

**Table 2:** Baseline characteristics.

	N	(%)	Mean(SD)
Overall	3506	(100.0)	
Sex	2105	(60.0)	
Male			
Female	1401	(40.0)	
Age (yrs)	3498		62.8(11.9)
BMI (kg/m^2^)	3506		23.7(3.3)
Weight (kg)	3506		61.8(11.6)
Duration of diabetes (yr)			
<1	44	(1.3)	
≥1, <5	406	(11.6)	
≥5	2856	(81.5)	
Unknown	200	(5.7)	
Number of pre-study OADs			
One	717	(20.5)	
Two	1434	(40.9)	
Three	1046	(29.8)	
Four or more	309	(8.8)	
Pre-study OADs			
Biguanides	1665	(47.5)	
Sulfonylureas	3118	(88.9)	
Glinides	249	(7.1)	
Alpha-glucosidase inhibitors	1621	(46.2)	
Thiazolidinediones	1216	(34.7)	
Neuropathy	873	(24.9)	
Retinopathy	907	(25.9)	
Nephropathy	891	(25.4)	
eGFR (mL/min/1.73 m^2^)	503	(14.3)	
<60			
≥60, <90	1292	(36.9)	
≥90	875	(25.0)	
Starting dose (U/kg/day)	3506		0.100(0.056)
Baseline HbA1c (%)	3515		9.53(1.19)

This study was conducted in conformity with the ethical principles of the Declaration of Helsinki, the Good Postmarketing Study Practice, and Good Vigilance Practice in Japan.

### Treatment, Follow-up, and Assessment

Concomitant OAD(s) and the starting and adjusted doses of insulin glargine were determined at physicians’ discretion. Safety and effectiveness data were collected over 24 weeks. Effectiveness parameters included an overall assessment of effectiveness by physicians, HbA1c, FPG, postprandial glucose (PPG), and weight. All AEs reported during the observational period were documented.

**Figure 3:**
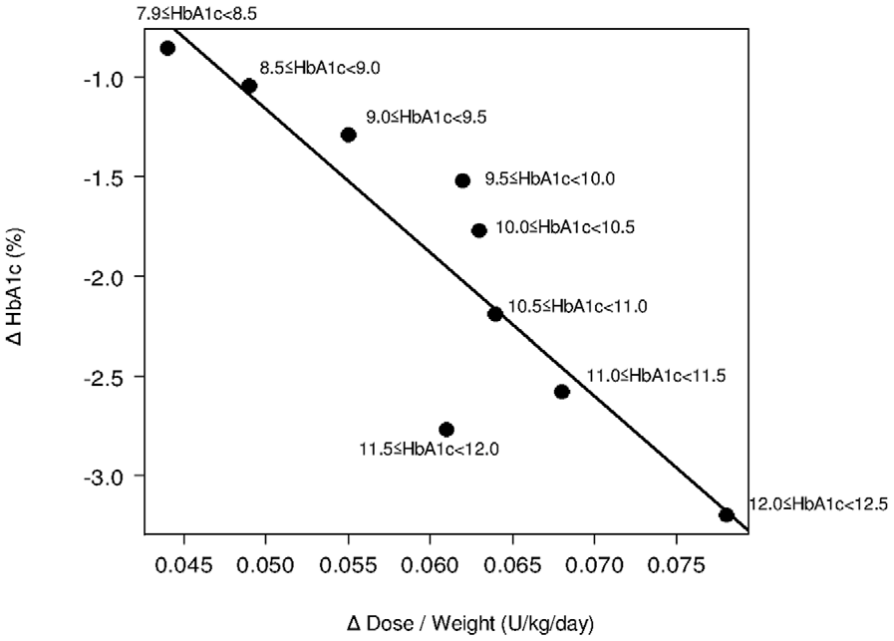
Plots and regression line describing the association between changes in dose per weight and HbA1c stratified by baseline HbA1c levels. r = −0.8900; Adjusted R^2^ = 0.7624 Δ HbA1c = −72.0845×Δ Dose/Weight+2.4449 Δ Dose = (−Δ HbA1c+2.4449) × Weight/72.0845.

### Statistical Analysis

Data from insulin naïve patients with available dose information from their final visit (24 weeks or at the end of observation) (n = 3506) were analyzed [Figure 1]. When patients did not complete the 24-week observational period, the last observed value was imputed based on the last-observation-carried-forward method.

**Table 3:** Starting dose per body weight and incremental dose coefficients for checking factors affecting starting and incremental doses.

			Starting dose(U/kg/day)	Differencefrom μ = 0.1501)	Differences in incremental dose coefficients among subgroups2)
		n	Mean±SD	P	P
Overall		182	0.142±0.103	n.s.	
Subgroups					
Sex	Men	122	0.140±0.112	n.s.	n.s.
	Women	97	0.135±0.070	*	
Age (yr)	<65	95	0.135±0.111	n.s.	n.s.
	≥65	86	0.151±0.093	n.s.	
Duration of diabetes (yr)	<5	29	0.143±0.085	n.s.	*
	≥5	138	0.143±0.106	n.s.	
BMI (kg/m^2^)	<24	100	0.148±0.078	n.s.	n.s.
	≥24	82	0.136±0.126	n.s.	
Body weight (kg)	<55	93	0.146±0.071	n.s.	n.s.
	≥55, <65	62	0.136±0.082	n.s.	
	≥65	63	0.136±0.133	n.s.	
Retinopathy	No	148	0.147±0.108	n.s.	n.s.
	Yes	34	0.120±0.072	*	
eGFR (mL/min/1.73 m^2^)	<60	48	0.114±0.062	**	n.s.
	≥60, <90	61	0.163±0.147	n.s.	
	≥90	42	0.141±0.082	n.s.	
Concomitant OADs	With sulfonylureas	133	0.132±0.100	*	n.s.
	With glinides	23	0.165±0.097	n.s.	
	Without secretagogues	20	0.187±0.122	n.s.	

Note: “Starting dose” indicates that of patients achieving the target HbAlc, which included those with HbAlc<7.0%, with optimal titration (average dose or more of that in the group achieving the target HbAlc).

1) one sample t-test for comparison with the ideal starting dose (μ = 0.150).

2) statistical test in the linear model with product terms between incremental dose and subgroup category.

n.s.: not significant, *P<0.05, **P<0.001.

**Figure 4:**
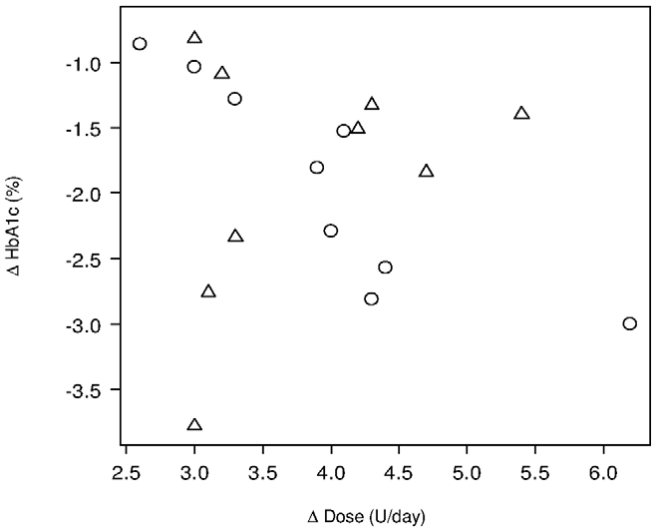
Effects of diabetes duration on the association between changes in dose and HbA1c stratified by HbA1c levels. Circles represent patients with a long duration of diabetes (≥5 years) and triangles means those with a short duration (<5 years). The long duration group showed a linear correlation, while there was no relationship in the short duration group.

Demographic characteristics were summarized as descriptive statistics. The mean change and SD for glargine doses and HbA1c levels were calculated according to baseline HbA1c strata. The change in dose by HbA1c was plotted according to the baseline HbA1c strata, and linear regression analysis was used to analyze the relationship between dose and HbA1c (n = 3506 for change in dose and n = 3187 for change in HbA1c) (Table 1 and Figure 2). The reliability of the regression line was examined using the split-half method [9]. We calculated the mean starting dose in 182 patients who achieved the target HbA1c (<7.0%) with the optimal glargine dose (above the average level [8.5 U/day] of the group achieving the target) at 24 weeks. Since the mean starting dose was 0.142 U/kg/day, we defined 0.15 U/kg/day as an optimal starting dose in this study population. This starting dose is more conservative than that commonly used in western populations (0.2 U/kg/day), and we assumed that the conservative dose of 0.15 U/kg/day was selected to minimize the risk of hypoglycemia in patients who might be highly insulin-sensitive [10]. This mean starting dose was also calculated based on subcategories and statistically compared with the optimal starting dose (μ = 0.150) using the one sample *t*-test. Regression lines between changes in HbA1c and dose were generated for each subcategory and coincident regressions were then examined for each category using linear models with product terms using average values of changes in dose and in HbA1c according to baseline HbA1c levels. The nomogram indicating the dose at 24 weeks needed to achieve HbA1c below 7% was constructed based on the formula generated in the current study.

**Figure 5:**
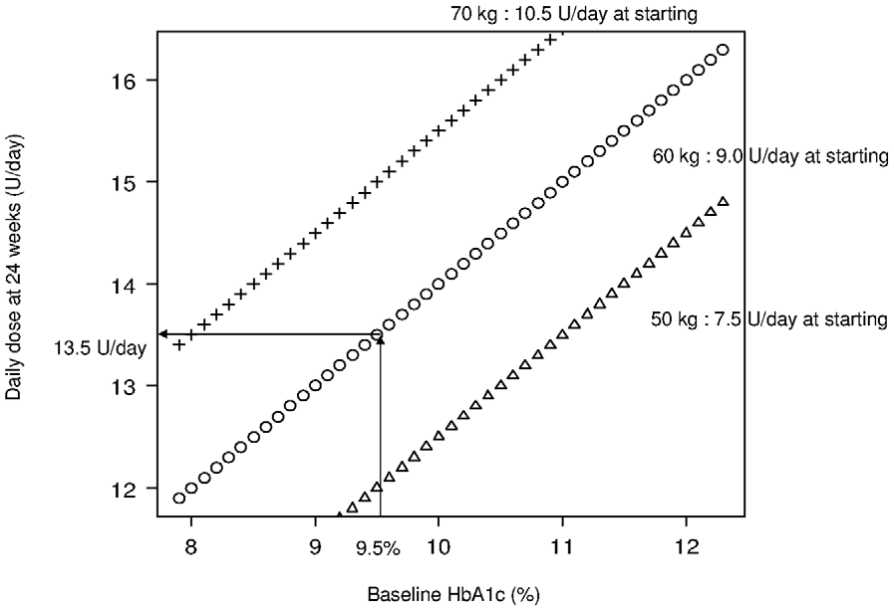
Nomogram of relationship between baseline HbA1c and daily dose at 24 weeks, targeting HbA1c<7.0%. Target HbA1c (<7.0%) was computed based on the following formula: Daily dose at 24 weeks (U/day) =  Starting dose + Incremental dose = 0.15×Weight + (Baseline HbA1c – Target HbA1c)+2.

All statistical tests were two-tailed and P<0.05 was considered to indicate statistical significance. Calculations were conducted using SAS System software, version 9.1.3 and R 2.13.1 [11].

## Results

### Baseline Characteristics

The baseline characteristics of 3506 insulin naïve patients whose dose was documented both at baseline and the final visit are summarized in Table 2. Mean±SD patient age was 62.8±11.9 years, and 60.0% were male. Mean BMI was 23.7±3.3 kg/m^2^, and 81.5% of patients had a disease duration of at least 5 years. As diabetic complications, neuropathy, retinopathy and nephropathy were documented in 24.9%, 25.9%, and 25.4% of patients, respectively. The mean initial glargine dose was 0.100±0.056 U/kg/day.

### Exploration of Incremental Dose Calculation

Changes in glargine dose and HbA1c, by baseline HbA1c, are shown in Table 1. As a whole, higher baseline HbA1c levels resulted in greater changes in the glargine dose and HbA1c. An inverse linear trend was observed between changes in HbA1c and dose (either U/day or U/kg/day) [Figures 2 and 3]. The correlation coefficient and the coefficient of determination were r = −0.9043 and adjusted R^2^ = 0.7918 for U/day and r = −0.8900 and adjusted R^2^ = 0.7624 for U/kg/day, respectively. Based on our aim of deriving a practical and simple formula for routine care, the following regression formula calculated with the U/day dose was employed: Δ dose = −0.964×Δ HbA1c+2.000, which was simplified as follows; the incremental dose at 24 weeks = − Δ HbA1c+2 =  (baseline HbA1c – target HbA1c)+2 (U/day). The split-half method was used to examine the reliability of the formula, and this evaluation revealed similar regression lines between the two randomly separated samples.

### Factors Affecting Starting and Incremental Doses

As shown in Table 3, a significantly reduced starting dose as compared to the optimal dose (0.15 U/kg/day) was prescribed for patients with eGFR below 60 mL/min/1.73 m^2^ (0.114 U/kg/day, P<0.001), those with retinopathy (0.120 U/kg/day, P<0.05), those with concomitant sulfonylurea use (0.132 U/kg/day, P<0.05), and women (0.135 U/kg/day, P<0.05). In checking the factors affecting incremental dose by the comparison of linear regression lines for subcategories, only duration of diabetes (<5 yrs and ≥5 yrs) was a statistically significant factor affecting the association between changes in dose and HbA1c. An inverse linear trend was observed among patients with a duration of diabetes ≥5 yrs while there was no trend among those with a duration <5 yrs [Figure 4].

### Examples of Dose Calculations

The recommended daily dose at 24 weeks (U/day) is calculated as follows; Starting dose (U/day) [0.15 (U/kg/day)×weight (kg)] + Incremental dose (U/day) (baseline HbA1c – target HbA1c+2) (baseline > target HbA1c). This formula can be described as a nomogram [Figure 5]. Three lines provide guidance for determining the daily glargine dose at 24 weeks based on baseline HbA1c and patient weight (for example, 50 kg, 60 kg and 70 kg). When a patient weighs 60 kg and HbA1c is 9.5% at baseline, the formula recommends that the patient be started on a dose of 9.0 U/day (60 kg×0.15 U/kg/day), which is then titrated by +4.5 U/day [(9.5–7.0)+2], to finally reach 13.5 U/day [9.0+(9.5–7.0)+2.0 U/day], in order to achieve HbA1c<7.0% at 24 weeks after starting glargine BOT.

### Hypoglycemic Rate

During the 24-week follow-up, 38 cases of hypoglycemia were observed in 3515 patients. Hypoglycemic rates stratified by baseline HbA1c levels were 1.3% for 7.9–8.4, 1.0% for 8.5–8.9, 0.8% for 9.0–9.4, 0.8% for 9.5–9.9, 1.5% for 10.0–10.4, 1.0% for 10.5–10.9, 2.0% for 11.0–11.4, 0.7% for 11.5–11.9, and 0.6% for 12.0–12.5.

## Discussion

A dose calculator for deriving the recommended glargine dose was generated for application to the clinical practice for Japanese type 2 diabetes patients who initiated insulin glargine after the treatment with OADs. Our dose calculation formula is expressed as: [optimal daily dose of insulin glargine at 24 weeks]  =  starting dose (0.15×weight) + incremental dose (baseline HbA1c – target HbA1c+2). It is noteworthy that the starting dose needs to be lower for some patient groups (those who have renal insufficiency, retinopathy, concomitant sulfonylurea use, and women).

The first major advantage of this formula is a good practicality in the Japanese clinical settings. Our formula contains HbA1c as a primary parameter: HbA1c is mostly measured as the best indicator of glycemic control in usual Japanese clinical settings. The American Diabetes Association and European Association for the Study of Diabetes released a recommended algorithm based on FPG-monitoring [2]. However, self-monitoring of FPG for the purpose of titration is not so widely penetrated in Japan as in western countries. Although FPG measurement is sometimes conducted at clinic visits in Japan, it may be avoided because of the increased risk of hypoglycemia when patients take medicines in fasting status on the visit day.

The second is the simplicity that can make the daily practice easier. The target doses are easily defined without complex calculations, leading to the reduction of the time burden on physicians. The simpler goal setting will also contribute to efficiency in patient education and empowerment. To our knowledge, our formula seems to be the simplest to calculate the optimal daily doses of insulin. The classical formula to estimate daily basal insulin doses was suggested by Holman and Turner, which is more complicated and based on the FPG level with weight correction [12].

We defined a starting dose of 0.15 U/kg/day as the “optimal initial dose” based on the results obtained from the patient subgroup achieving HbA1c levels below 7.0%. Compared to previous studies, this dose would be rather conservative [10]. A German observational study of glargine BOT found the average initial dose to be approximately 0.17 U/kg/day [6]. Another comparable investigation, the UK THIN database study, found the initial glargine dose to be 0.4 U/kg/day in patients with a mean HbA1c of 9.5% at baseline, among whom 30% achieved HbA1c ≤7.5% at 12 months [7]. Based on these results, starting doses appear to differ among various clinical populations. Further studies are needed to explain the reason why these differences occur.

Our dose calculation formula may have several limitations. First, it is not always applicable to any patients with type 2 diabetes. This formula was generated using data from insulin naïve patients who had already been administered OADs and had baseline HbA1c of 8% to 12%. Furthermore, the optimal starting dose (0.15 U/kg/day) was derived from a portion of our sample, i.e., those who achieved the target HbA1c with a substantial dose increase. In addition, patients documented in the ALOHA study were generally not obese (mean BMI of 23.7 kg/m^2^) as compared with many other studies, especially those of non-Asian populations [13]. Therefore, our findings may well be more applicable to non-obese populations, such as those of Asia. If our formula is applied to obese populations, another version using U/kg/day to determine doses (Figure 3) would likely be more appropriate.

Second, although one of the predictive factors contributing to the achievement of the target HbA1c was the starting dose, other factors such as severity of diabetes may also contribute to such outcomes. In fact, another ALOHA subanalysis revealed that lower HbA1c levels, shorter diabetes duration, and absence of retinopathy were associated with the achievement of HbA1c<7.0% (unpublished data). However, we confirmed the homogeneity across patients with characteristics potentially associated with the target achievement (Table 3).

Finally, the “optimal” dose, which we assumed, may be “suboptimal” for some patients. Both starting and incremental doses were derived from a population not based on the Treat-to-Target approach trial but based on the target HbA1c achievers who occupy less than 20% of all patients in ALOHA study. Thus, this formula may underestimate the dose needed to achieve the target HbA1c.

In conclusion, we derived the following dose calculation formula: optimal daily dose at 24 weeks  =  starting dose (0.15×weight) + incremental dose (baseline HbA1c – target HbA1c+2). This formula is simple, practical, and potentially applicable to most Japanese type 2 diabetes patients starting glargine BOT. The starting dose needs to have a careful attention for some patient groups; i.e., those who have renal insufficiency, retinopathy, concomitant sulfonylurea use, and women.
