# Correction: Potential Formula for the Calculation of Starting and Incremental Insulin Glargine Doses: ALOHA Subanalysis

**DOI:** 10.1371/journal.pone.0103390

**Published:** 2014-07-18

**Authors:** The PLOS ONE Staff

The role of the funder is stated incorrectly in the Financial Disclosure (FD) statement of this article. Please refer to the correct FD statement below:

This study was sponsored by sanofi-aventis K.K. Dr. Tetsuya Ohtani is an employee of sanofi-aventis KK and received salary from sanofi-aventis KK. The funder had a role in study design, data collection and analysis, decision to publish, and preparation of the manuscript.
