# Correction: Potential Formula for the Calculation of Starting and Incremental Insulin Glargine Doses: ALOHA Subanalysis

**DOI:** 10.1371/journal.pone.0106273

**Published:** 2014-08-18

**Authors:** The PLOS ONE Staff

Dr. Yusuke Naito is the third author and also a corresponding author for this article. His affiliation is:

^2^Sanofi-aventis K.K., Tokyo, Japan. His contributions are as follows: analyzed the data and wrote the paper.

The correct citation and contact information of the corresponding authors are:

Kadowaki T, Ohtani T, Naito Y, Odawara M (2012) Potential Formula for the Calculation of Starting and Incremental Insulin Glargine Doses: ALOHA Subanalysis. PLoS ONE 7(8): e41358. doi:10.1371/journal.pone.0041358

Corresponding Authors

Takashi Kadowaki (kadowaki-3im@h.u-tokyo.ac.jp)

Yusuke Naito (yusuke.naito@sanofi.com)
